# Diagnostic Concordance in Tertiary (Dermatologists-to-Experts) Teledermoscopy: A Final Diagnosis-Based Study on 290 Cases

**DOI:** 10.5826/dpc.1003a71

**Published:** 2020-06-29

**Authors:** Anne Marchetti, Stephane Dalle, Delphine Maucort-Boulch, Mona Amini-Adl, Sébastien Debarbieux, Nicolas Poulalhon, Marie Perier-Muzet, Alice Phan, Luc Thomas

**Affiliations:** 1 Service de Dermatologie, Centre Hospitalier Lyon Sud, France; 2 Service de Biostatistique-Bioinformatique, Pôle Santé Publique, Hospices Civils de Lyon, France; 3 Laboratoire de Biométrie et Biologie Évolutive, Équipe Biostatistique-Santé, Villeurbanne, France; 4 Université de Lyon, Lyon, France; 5 Université Claude Bernard, Lyon, France; 6 Centre de Recherche en Cancérologie de Lyon, France

**Keywords:** teledermoscopy, tertiary teledermatology, nail, pediatric, dermoscopy

## Abstract

**Background:**

Teledermoscopy (TDS) improves diagnostic accuracy and decreases the number of unnecessary consultations.

**Objectives:**

To determine the diagnostic concordance in tertiary (dermatologist-to-experts) TDS with histopathology/follow-up–based diagnosis.

**Methods:**

A descriptive retrospective cohort study including 290 requests.

**Results:**

Perfect diagnostic concordance was found in 202 (69.7%) cases and partial agreement in 29 (10%). Disagreement was found in 59 (20.3%) cases. Perfect concordance on the benign/malignant nature of the lesion was found in 227 (78.3%) cases and disagreement in 63 (21.7%). In onychology, diagnostic concordance was perfect in 43 (76.8%) cases, partial in 7 (12.5%), and there was disagreement in 6 (10.7%). Final concordance on the benign/malignant nature of the lesion was perfect in 48 (85.7%) and there was disagreement in 8 (14.3%) nail cases. For pediatric requests, diagnostic concordance was perfect in 29 (65.9%) cases, partial in 5 (11.4%), and there was disagreement in 10 (22.7%). Final concordance on the benign/malignant nature of the lesion was observed in 34 (77.3%) cases, disagreement in 10 (22.7%).

**Conclusions:**

This study confirms that tertiary TDS improves diagnostic accuracy of pigmented skin lesions. Moreover, it shows encouraging results in unusual conditions such as ungual and pediatric skin tumors. The main limitation was the retrospective nature and the “real-life” setting of our study that could have created a selection bias toward inclusion of the most difficult cases.

## Introduction

Diagnosis of skin cancer is challenging for solitary practicing dermatologists. Teledermatology (TD) exists in 2 modes: store-and-forward and live interactive [1]; in store-and-forward, the most used, information is sent to an electronic platform for delayed analysis. In 2 systematic reviews, TD was found inferior to face-to-face (FTF) dermatology but the accuracy was deemed acceptable by the authors [2,3]. However, Coates et al pointed out some limitations of TD such as the lack of total-body skin examinations [4].

Teledermatology is classified as primary, secondary, and tertiary TD. Primary TD involves communication between patients and a general practitioner (GP). In secondary TD, GPs communicate with dermatologists. In tertiary TD, dermatologists receive an expert opinion [1]. According to Finnane et al, the main limitation of all TD studies published since 2009 was that tele-expert diagnosis was compared to that of a primary physician, not to the final histopathology/follow-up–based diagnosis [2]. Teledermoscopy (TDS) is based on transmission of a dermoscopy picture.

TDS is known to improve diagnostic accuracy and decrease the rate of unnecessary consultations in dermatology compared with TD alone [5–7], yet most of the published studies were performed in a secondary telemedicine setting. By contrast, the aim of this study, performed in our unit dedicated to private practice dermatologists with special extra-competence in difficult-to-diagnose skin lesions encompassing many digital dermoscopy follow-ups, nail tumors, and pediatric lesions, was to (1) determine the final diagnostic concordance between the diagnosis made by the tele-expert and the final diagnosis and (2) evaluate the efficiency of tertiary TDS.

## Methods

### Patients

We conducted an unselected consecutive cohort study between January 1, 2016, and December 31, 2016. Referring clinicians sent TD requests on an encrypted, firewall-protected store-and-forward server of the Hospices Civils de Lyon (https://myhclpro.sante-ra.fr/). Clinicians provided age, sex, location, personal and family medical history, and macroscopic and dermoscopic pictures to experts and questions on diagnosis and management. We restricted this study to dermoscopy containing pictures requests made by dermatologists or skin cancer hyperspecialized GPs. In cases of multiple TD consultations for the same lesion, only the first was considered, whereas in requests for multiple lesions, each lesion was analyzed as a single statistical event. In this “real-life” study, no standardization of picturing mode was used. These pictures were analyzed by 1 among the 7 experts (at least 10 years of practice of dermoscopy and 10 publications on the field of dermoscopy) in TDS in the department; response was sent to the referring clinician and saved for analysis. Experts responded about diagnosis, possible differential diagnoses, the benign/malignant nature of the lesion, and management (excision, follow-up, biopsy, confocal microscopy, or picturing).

### Case Revisions

All pictures and answers have been retrospectively analyzed by a dermatology resident (A.M.) and a senior expert (S.D., L.T.). Lesions were subclassified into pigmented skin lesions (PSL), amelanotic tumors (AT), nail pigmentation (NP), and other nail lesions (NL). Picture quality was assessed.

The gold standard was either histopathology or reasonable-delay (at least 1 year) benign evolution in all cases. Indeed, the level of evidence is weaker in the second case. However, systematic surgical excision of all cases would have been unethical. Moreover, surgical recommendation for benign conditions to an expert’s eyes would have resulted in greater bias since our series could not then be considered a “real-life” one. Cases with neither histopathological nor follow-up information were excluded.

The large number of pediatric and onychology cases allowed us to perform a subgroup analysis.

### Definitions of Concordance/Disagreement

The definitions of concordance and disagreement are indicated in Table 1.

### Statistical Analysis

Analyzes were done with R software (version 3.4.4, R Development Core Team. R: A Language Environment for Statistical Computing. Vienna, Austria. ISBN 3-900051-07-0. URL: http://www.R-project.org,2018) by an independent statistics expert (D.M.B.). We used average, standard deviation, median, first and third quartile, range for continuous variables; and percentages and effectives for discontinuous variables. Variables were compared with chi-square or Fischer exact test when necessary.

The Ethical Committee of Lyons University Hospital approved the study protocol on May 17, 2018.

## Results

### Populations

**Figure 1:**
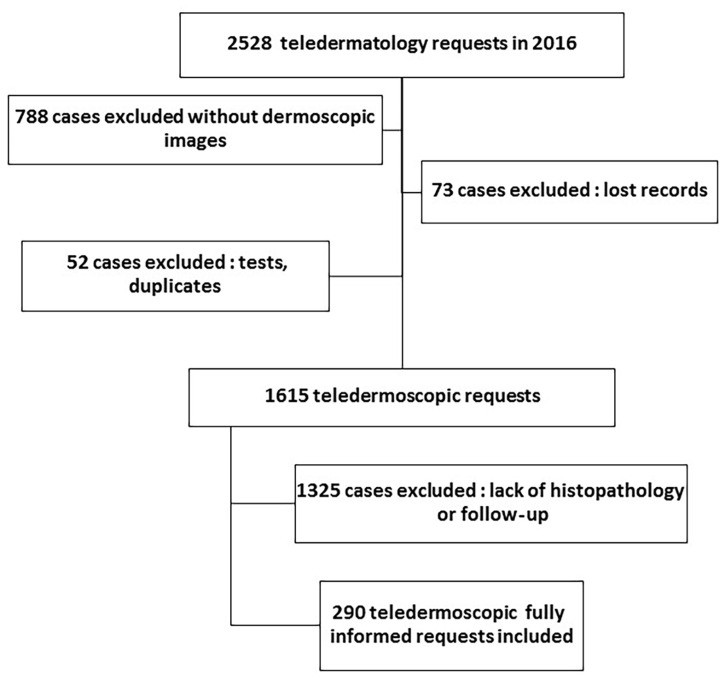
Flow chart of the study.

Figure 1 represents the flow chart of the study. Two hundred ninety teledermoscopic requests with known final diagnosis were included on a total of 2,528 tertiary TD requests sent between January 1, 2016, and December 31, 2016. One hundred seventy-seven (61%) patients were female and 113 (39%) male; median age was 45 years and there were 44 (15.2%) children (aged ≤15 years).

### Referring Clinicians

Referring clinicians were 78 (93.9%) dermatologists and 6 (7.1 %) skin cancer hyperspecialized GPs. Referring clinicians of our geographic region (Rhône Alpes Auvergne) accounted for 152 (53.9%); 2 (0.7%) requests were international.

### Requests

Requests included a median number of 2 (range 1–6) dermoscopy pictures in a total number of 3 (range 1–24). In 32 cases (11%) we did not receive accompanying close-up or wide-angle standard pictures. These standard images were present in 258 (89%) cases. Dermoscopy pictures were of good quality in 260 (89.7%) cases. In 170 (58.6%) cases, a report on medical history of the patient was lacking. A personal history of melanoma was mentioned in 25 (8.6%) cases. Family history of melanoma was reported in 14 (4.8%) cases. The most common purpose for TDS consultation was evaluation of PSL in 199 (68.6%) cases, followed by nail diseases in 56 (19.3%) cases, then by AT in 35 (12.1%). Referring forms reported history of an enlarging lesion in 33 (11.4%) cases, change in a preexisting lesion in 11 (3.8%) and onset of a new lesion in 56 (19.3%). The evolution time before teleconsultation was 6 to 12 months in 55 (19%) cases; only 14 (4.8%) lesions were present for less than 3 months. Diagnosis was the principal question in 42 (14.5%) cases, then management in 183 (63.1%) and both in 48 (16.5%). In 32 (11%) cases, motivation was to obtain a university hospital appointment (e-referral).

### Expert Answers

Experts submitted their answers in a mean time of 2.21 days (median 1 day; range 1–14 days). In 11 (3.8%) cases, no diagnosis was made by an expert. One expert answered to 177 (61%) requests and to 41 (73.2%) nail requests. Pediatric cases were managed by one expert with acknowledged hyperspecialization in pediatric dermatology (A.P.) in 19 (43.2%) and by another expert in 18 (40.9 %). Onychology represented 7 (15.9%) pediatric cases.

### Main Diagnoses

All diagnoses are reported in Table 2. The most common diagnoses for PSL were benign melanocytic lesion in 134 (67.3%) cases, ungual squamous cell carcinoma in 10 (35.7%) for NL (NP excluded), focal melanocytic activation in 10 (35.7%) cases of NP, and basal cell carcinoma for AT in 8 (22.9%) cases.

### Management of Skin Tumors and PSL

Excision was recommended in 65/290 (22.4%) of all skin tumors and in 44/199 (21.1%) of PSL; 3-month follow-up was recommended in 50 (17.4%); nail biopsy was recommended in 26 (46.4%) nail cases. When recommended, biopsies and excisions were performed in all cases. Experts recommended a hyperspecialized university hospital consultation in 65 (22.6%) cases; they considered it unnecessary in 2 among 32 cases for whom it was requested (6.3%).

### Diagnostic Correlation

**Figure 2:**
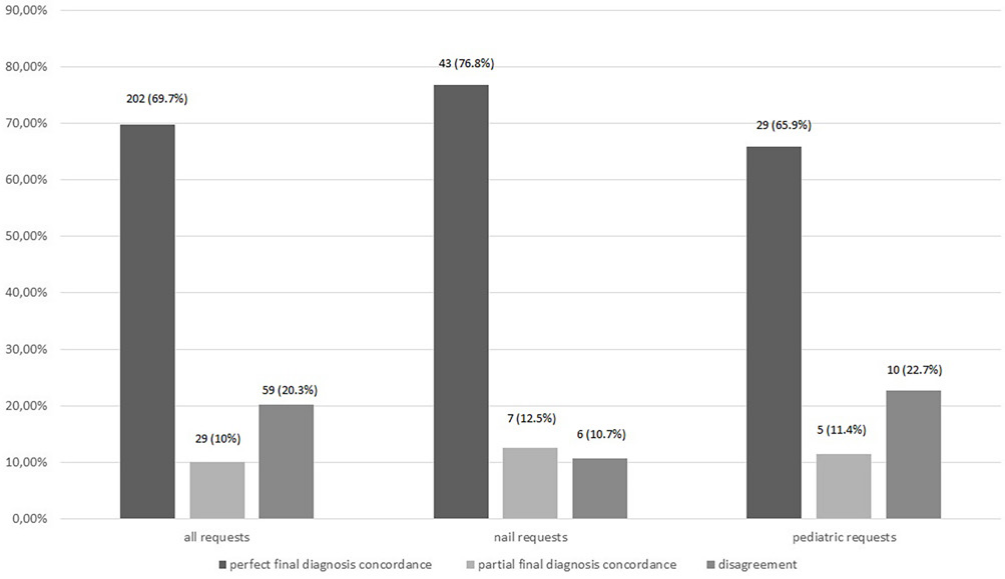
Perfect final diagnostic concordance between teledermoscopy expert and final diagnoses (histopathology or reasonable-delay benign follow-up).

**Figure 3:**
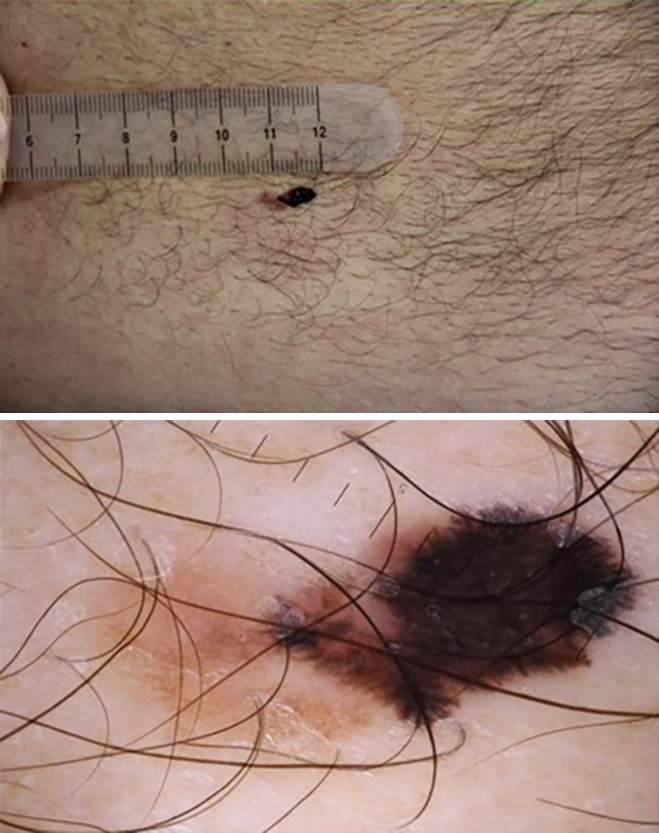
A 72-year-old man presented with a pigmented atypical lesion on the abdomen for 6 months. Expert and clinicians both diagnosed a melanoma and suggested excision of the lesion. Histology found a superficial spreading melanoma 0.35 mm thick.

**Figure 4:**
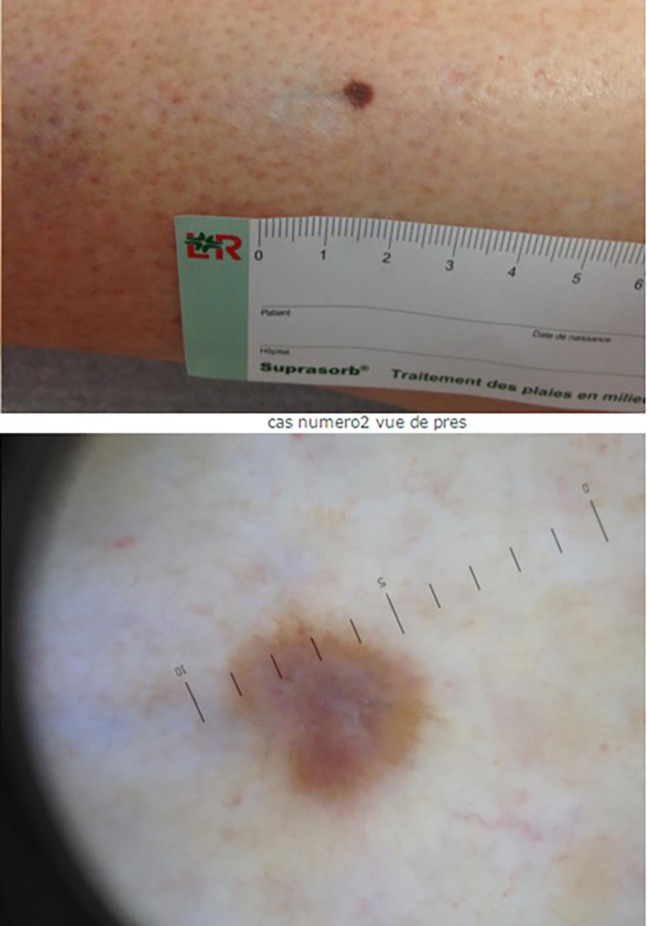
A 70-year-old woman presented with a pigmented atypical lesion on the leg. The referring clinician suggested excision for a possible melanoma. The diagnosis of dermatofibroma, suggested by the expert, was confirmed by histopathology.

Histopathological diagnosis was available in 167 (57.6%) cases and reasonable-delay benign follow-up in the remaining 123 (42.4%). Perfect final diagnostic concordance between teledermatologists and histopathology or follow-up was found in 202 (69.7%) cases (Figure 2). Partial concordance was found in an additional 29 (10%) cases. Disagreement was found in 59 (20.3%) cases; in 51 (86.4%), a benign lesion for which management was not compromised was found. Among the remaining cases, 2 were melanomas: 1 was left undiagnosed because of a poor-quality picture (and reported as such to the referring clinician and finally excised) and the other was a 0.2-mm melanoma diagnosed as an atypical nevus for which a 6-month follow-up was suggested, which then led to the correct diagnosis. Other misdiagnosed tumors were 5 basal cell carcinomas (in 3 cases histopathology was, however, recommended; in 1 case a 3-month follow-up was recommended; no treatment was recommended in the fifth case) and 1 squamous cell carcinoma (no response because of poor-quality picture). Prediagnostic concordance is presented in Table 3. For example, the referring clinician and expert totally agreed on the diagnosis of the histopathology-confirmed melanoma shown in Figure 3. However, the referring clinician and expert disagreed on diagnosis and management of the lesion shown in Figure 4 but histopathology confirmed a dermatofibroma, as proposed by the expert.

**Figure 5:**
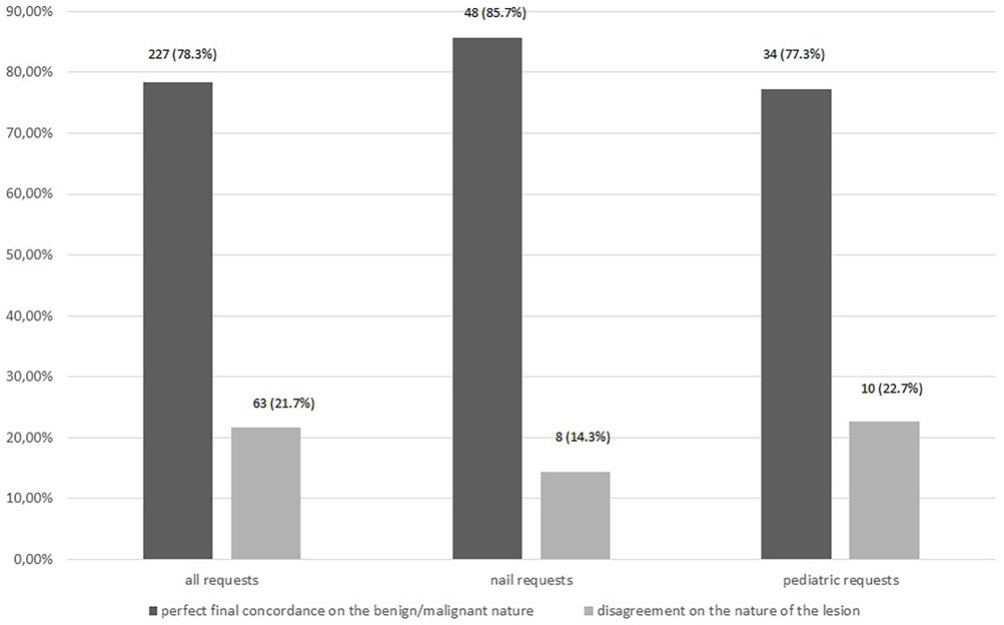
Perfect final concordance on the benign/malignant nature of the lesion between teledermoscopy expert and final diagnoses (histopathology or reasonable-delay benign follow-up).

Benign/malignant concordance was found in 227 (78.3%) cases, discordance in 63 (21.7%) cases (Figure 5). Prediagnostic benign/malignant concordance and management concordance results are presented in Tables 4 and 5, respectively. Results in the nail and pediatric subgroups are presented in Figures 2 and 5 and Tables 3, 4, and 5.

The experts’ diagnostic concordance with final diagnosis was statistically lower for AT: total disagreement on 18/35 (51.4%) when compared to 57/199 (28.6%); 6/28 (21.4%) and 7/28 (25%) for PSL, NP, and NL (P = 0.028), respectively.

## Discussion

We report herein the first robust, final diagnosis-based, real-life concordance study in tertiary (specialists-to-experts) store-and-forward TDS.

Tertiary TD is used in order to seek expert opinion/second opinion, but also to obtain an expert FTF consultation (e-referral). It may also be used for resident training and continuous medical education of specialists [8]. TDS is a specialized approach within TD known to improve diagnostic accuracy and to decrease the rate of unnecessary consultations in dermatology compared with TD without dermoscopy [5–7], yet all published studies to date were performed in a secondary telemedicine setting.

In most published TD studies, the main methodological limitation was the absence of correlation study with final diagnosis established either on histopathology or follow-up [2]. Moreover, no secondary-setting TDS published studies included follow-up information for nonexcised lesions [2]. In our study, the gold standard was histopathology in 57.6% of the cases or follow-up in 42.4%. We report a high (79.7%) diagnostic concordance between TDS experts and final diagnosis except for diagnosis of AT, for which the diagnostic discordance was significantly higher (51%) than for PSL (28.6%), NL (25%), and NP (21.4%). We also report a high (78.3%) concordance level about the diagnosis of malignant/benign nature of the lesion. Our results suggest that TDS improved diagnosis and management because of an observed high level of discordance between diagnosis proposed by the referring clinician and the expert (44.8%) and a high frequency of alternative management proposed by the expert (46.5%). Interestingly enough, analysis of our misdiagnosed cases showed only 1/23 (4.3%) undiagnosed melanoma and concerned an early case (0.2 mm) for which digital follow-up was suggested. Improvement on the diagnosis regarding the malignant/benign nature of the referred lesion is also suggested by a 45.2% prediagnostic benign/malignant disagreement. Moreover, analysis of diagnostic disagreements between expert and referring clinician showed that the expert was correct in the majority of cases (72.7%). Our results in tertiary TDS are similar to previously published results in secondary settings (51%–94% for diagnostic accuracy between TD and histopathology [excised lesion]) and FTF diagnosis (nonexcised lesions) when dermoscopy is performed [2,9–17]. Literature data combined with ours suggest an interesting improvement of diagnosis by transmission of dermoscopy pictures in cases of doubtful PSL. However, one author reported that the addition of TDS did not significantly improve diagnostic accuracy compared to transmission of standard pictures alone for malignant PSL [18] and suggested that TDS was useful only for malignant amelanotic skin tumors (aggregated accuracy, P = 0.0017; primary accuracy, P = 0.0382) [19]. This discrepancy might be explained by the tertiary setting of our study, referred cases being found difficult-to-diagnose by dermatology specialists concerned less with basal cell carcinomas and more with unusual (acral, pediatric) pigmented skin tumors.

The only available report on histopathology-based tertiary TD, including 33 cases, was centered on inflammatory skin diseases; it also demonstrated a high (78.8%) level of concordance [20]. Van der Heijden et al showed that in 81% of cases, dermatologists would have referred the patient to a tertiary center without the help of TD [21]. Our study also demonstrates that, in “real life,” the delay to obtain an expert opinion is short (2.21 days, median 1 day). In our study, 65 (22.6%) cases were referred to our hospital after TDS. Although almost half of our TDS recruitment is generated outside our region, this high number is explained by the high proportion of nail conditions for which biopsy is difficult to conduct in home offices. Cheung et al also demonstrated that TD avoided FTF consultation in 68% of cases with good-quality pictures [22]. Dermoscopy picture quality was good in 89.7% of cases in our study, similar to Massone et al (88%) [14]. In another secondary-setting TDS study, the quality of transmitted pictures was relatively lower, with only 36% good and 28% fair [12] and a lower accuracy and reliability compared with FTF consultation (0.66 on diagnosis and 0.42 on management), supporting the concept that TDS is highly dependent on picture quality [12].

Our study assessed the concordance between TDS and final diagnosis in pediatric skin tumors. Consultation offers in pediatric dermatology are scarce and are by far exceeded by the demand. TD is used to facilitate access to highly specialized pediatric dermatology opinion and to select cases for whom an FTF consultation is needed [23]. In the literature, diagnostic concordance between pediatricians and teledermatologists ranges from 16.7% to 82% [23–26]. These studies concern inflammatory skin diseases and do not include dermoscopy pictures. Diagnostic concordance was reasonably high (77.3%). Management concordance level between pediatricians and teledermatologists ranged from 25% to 44% vs 55% to 76% in adult patients. Our study concerned exclusively pediatric skin tumors, including dermoscopy pictures, and our results (59%) are very similar.

Our study encompassed a large number (19.3%) of nail teleconsultations. We found a high diagnostic concordance between TDS expert and final diagnosis in 50 (89.3%) and a high number of diagnostic (50%) and management (42.9%) changes after TDS, suggesting a high impact on patient’s outcome.

The main limitation of our work was its cohort nature and the “real-life” setting evaluating our routine telemedicine platform with no previously agreed-upon way to report on outcome. Many cases (89.5%) were excluded because of unavailable histopathology/outcome information. This could have created a selection bias toward inclusion of the most difficult cases, the referring clinicians being less willing to retrieve patient information in case of indolent conditions or unchanged diagnosis and management after TDS.

## Conclusions

In addition to previously published reports, this study confirms that tertiary TDS improves diagnostic accuracy of PSL compared with solitary nonexpert assessment and offers additional support for the management of unusual conditions such as ungual and pediatric skin tumors with easier access to regional, national, and international expert opinion.

## Figures and Tables

**Table 1:** Definitions of Study Outcomes

Study Outcome	Reference Standard	Index Test
Final diagnostic concordance	Histopathological result (excised or biopsied lesions) or follow-up (nonexcised lesions)	Teledermatologist expert diagnosis
Perfect final diagnostic concordance	Teledermatologists and final diagnosis is identical	
Partial concordance	Final diagnosis is included in the differential diagnosis list by the expert but not in first position	
Disagreement on final diagnosis	Final diagnosis not suggested by the expert in his differential diagnosis list	
Prediagnostic concordance	Teledermatologist diagnosis	Referring clinician proposed diagnosis
Perfect prediagnostic concordance	Teledermatologists and referring clinical initial proposition for diagnosis are the identical	
Partial prediagnostic concordance	Diagnosis given by the expert is included in the differential diagnoses list by the referring clinician but not in first position	
Disagreement on prediagnosis	Diagnosis given by the expert is different from the initially proposed one(s) by the referring clinician	
Management concordance	Teledermatologist management	Referring clinician proposed management
Perfect management concordance	Teledermatologists and referring clinical initial proposition for management are identical	
Partial concordance on management	Final management suggested by the expert is proposed by the referring clinician but not in first position	
Disagreement on management	The final management proposed by the expert is different from the initially proposed one by the referring clinician	
Benign/malignant concordance	Histopathological result or follow-up (nonexcised lesions)	Teledermatologist expert diagnosis on benign/malignant nature
Perfect final concordance on the benign/malignant nature of the lesion	Teledermatologists and final diagnosis on the benign/malignant nature of the lesion is identical	
Disagreement on the nature of the lesion	Teledermatologists and final diagnosis on the benign/malignant nature of the lesion is different	
Prediagnostic benign/malignant concordance	Teledermatologist diagnosis on benign/malignant nature	Referring clinician proposed diagnosis on benign/malignant nature
Perfect prediagnostic concordance on the benign/malignant nature of the lesion	Teledermatologists and referring clinical initial proposition for the benign/malignant nature of the lesion is identical	
Disagreement on prediagnosis on the nature of the lesion	Teledermatologists and referring clinical initial proposition for the benign/malignant nature of the lesion is different	

**Table 2:** Summary of All Teledermoscopy Final (Histopathology or Evolution) Diagnoses

Lesion Type	Main Diagnoses	Final Diagnoses (N = 290) (100%)	Final Pediatric diagnoses (n = 44) (15.2%)

Pigmented skin lesions	Benign melanocytic lesions (nevi, blue nevi, hallo nevi, congenital nevi)	134 (67.3%)	25 (83.3%)
n = 199 (68.6%)	Melanoma	17 (8.5%)	2 (6.7%)
n = 30 (15.1%)	Spitz tumors (Spitz nevi and malignant spitzoid tumors)	3 (1.5%)	2 (6.7%)
pediatric	Malignant epithelial tumors (BCC, Bowen disease, SCC)	7 (3.5%)	
	Benign epithelial tumors (seborrheic keratoses, lentigos)	31 (15.7%)	
	Other diagnoses		
	• Collision tumors	1 (0.5%)	
	• Dermatofibroma	3 (1.5%)	
	• Postinflammatory pigmentation	1 (0.5%)	
	• Exogenous pigmentation	1 (0.5%)	
	• Mastocytosis	1 (0.5%)	1 (3.3%)

Amelanotic tumors	Malignant epithelial tumors (BCC, Bowen disease, SCC)	11 (31.5%)	
n = 35 (12%)	Benign epithelial tumors (seborrheic keratoses, epidermoid cysts, warts)	4 (11.4%)	1 (14.3%)
n = 7 (20%)	Benign melanocytic lesions	4 (11.4%)	
pediatric	Spitz tumors (Spitz nevi and malignant spitzoid tumors)	2 (5.7%)	2 (28.6%)
	Melanoma	1 (2.9%)	
	Vascular lesions (angioma, pyogenic granuloma)	4 (11.4%)	
	Other diagnoses		
	• Dermatofibroma	3 (8.6%)	1 (14.3%)
	• Juvenile xanthogranuloma	2 (5.7%)	2 (28.6%)
	• Adnexal tumors (trichoblastoma, pilomatricoma)	2 (5.7%)	1 (14.2%)
	• Inflammatory diseases	2 (5.7%)	

Nail pathology (longitudinal NP excluded)	Subungual SCC	10 (35.7%)	
	Epithelial benign tumors (warts, onychopapilloma)	4 (14.2%)	
	Subungual exostosis	3 (10.7%)	1 (100%)
n = 28 (9.7%)	Melanoma	1 (3.6%)	
n = 1 (3.6%)	Other diagnoses		
pediatric	• Onychotillomania	1 (3.6%)	
	• Glomus tumor	1 (3.6%)	
	• Hamartoma	1 (3.6%)	
	• Trauma-induced nail changes	1 (3.6%)	
	• Onychomycosis	2 (7.1%)	
	• Pyogenic granuloma	2 (7.1%)	
	• Fibromyxoma	1 (3.6%)	
	• Myxoid pseudocyst	1 (3.6%)	

Longitudinal NP	Focal melanocytic activation including drug-induced NP, trauma-induced NP, ethnic-type NP	10 (35.7%)	1 (16.7%)
n = 28 (9.7%)			
n = 6 (21.4%)	Acquired benign melanocytic lesions	7 (25%)	2 (33.3%)
pediatric	Congenital nevi of the nail unit	3 (10.7%)	3 (50%)
	Melanoma	4 (14.3%)	
	Other diagnoses		
	• Subungual hemorrhage	1 (3.6%)	
	• Onychopapilloma	1 (3.6%)	
	• SCC	2 (7.1%)	

BCC = basal cell carcinoma; NP = nail pigmentation; SCC = squamous cell carcinoma.

**Table 3:** Prediagnostic Concordance Between Teledermoscopy Expert and Referring Clinician

	All Requests (N = 290) (100%)	Nail Requests (n = 56) (19.3%)	Pediatric Requests (n = 44) (15.2%)

Perfect prediagnostic concordance	116 (40%)	16 (28.6%)	21 (47.7%)

Partial prediagnostic concordance	44 (15.2%)	12 (21.4%)	4 (9.1%)

Disagreement on prediagnosis	130 (44.8%)	28 (50%)	19 (43.2%)
No hypothesis from referring clinician	76 (26.2%)	21 (37.5%)	9 (20.5%)
No hypothesis from teledermoscopy expert	11 (3.8%)	0	3 (6.8%)

**Table 4:** Management Concordance Between Teledermoscopy Expert and Referring Clinician

	All Requests (N = 290) (100%)	Nail Requests (n = 56) (19.3%)	Pediatric Requests (n = 44) (15.2%)

Perfect management concordance	75 (25.9%)	19 (33.9%)	13 (29.5%)

Partial concordance on management	80 (27.6%)	13 (23.2%)	13 (29.5%)

Disagreement on management	135 (46.5%)	24 (42.9%)	18 (41%)
No management proposed by the referring clinician	75 (25.9%)	15 (26.8%)	5 (11.4%)
No management proposed by expert in teledermoscopy	11 (3.8%)	0	0

**Table 5:** Prediagnostic Benign/Malignant Concordance Between Teledermoscopy Expert and Referring Clinician

	All Requests (N = 290) (100%)	Nail Requests (n = 56) (19.3%)	Pediatric Requests (n = 44) (15.2%)

Perfect prediagnostic concordance on benign/malignant nature of the lesion	159 (54.8%)	26 (46.4%)	29 (65.9%)

Disagreement on prediagnosis on the nature of the lesion	131 (45.2%)	30 (53.6%)	15 (34.1%)
No hypothesis from the referring clinicians	76 (26.2%)	21 (37.5%)	9 (20.5%)
No hypothesis from teledermoscopy expert	11 (3.8%)	0	3 (6.8%)
